# Increasing Transfers-Out from an Antiretroviral Treatment Service in South Africa: Patient Characteristics and Rates of Virological Non-Suppression

**DOI:** 10.1371/journal.pone.0057907

**Published:** 2013-03-05

**Authors:** Mweete D. Nglazi, Richard Kaplan, Catherine Orrell, Landon Myer, Robin Wood, Linda-Gail Bekker, Stephen D. Lawn

**Affiliations:** 1 The Desmond Tutu HIV Centre, Institute for Infectious Disease and Molecular Medicine, and the Department of Medicine, Faculty of Health Sciences, University of Cape Town, Cape Town, South Africa; 2 International Union against Tuberculosis and Lung Disease, Paris, France; 3 School of Public Health and Family Medicine, Faculty of Health Sciences, University of Cape Town, Cape Town, South Africa; 4 Department of Clinical Research, Faculty of Infectious and Tropical Diseases, London School of Hygiene & Tropical Medicine, London, United Kingdom; Boston University, United States of America

## Abstract

**Objectives:**

To determine the proportion, characteristics and outcomes of patients who transfer-out from an antiretroviral therapy (ART) service in a South African township.

**Methods:**

This retrospective cohort study included all patients aged ≥15 years who enrolled between September 2002 and December 2009. Follow-up data were censored in December 2010. Kaplan-Meier survival analysis was used to describe time to transfer-out and cox proportional hazard analysis was used to determine associated risk factors.

**Results:**

4511 patients (4003 ART-naïve and 508 non-naïve at baseline) received ART during the study period. Overall, 597 (13.2%) transferred out. The probability of transferring out by one year of ART steadily increased from 1.4% in 2002/2004 cohort to 8.9% for the 2009 cohort. Independent risk factors for transfer-out were more recent calendar year of enrolment, younger age (≤25 years) and being ART non-naïve at baseline (i.e., having previously transferred into this clinic from another facility). The proportions of patients transferred out who had a CD4 cell count <200 cells/µL and/or a viral load ≥1000 copies/mL were 19% and 20%, respectively.

**Conclusions:**

With scale-up of ART over time, an increasing proportion of patients are transferring between ART services and information systems are needed to track patients. Approximately one-fifth of these have viral loads >1000 copies/mL around the time of transfer, suggesting the need for careful adherence counseling and assessment of medication supplies among those planning transfer.

## Introduction

Increasing numbers of patients are receiving antiretroviral therapy (ART) at health facilities in sub-Saharan Africa and the World Health Organization (WHO) estimated that 5.1 million people had started ART in the region by the end of 2010. Of these, 1.4 million were in South Africa alone [1]. However, as scale-up has progressed, it has been reported that growing proportions of patients are not retained long-term within ART services with losses estimated at approximately 40% of patients during the first 2 years of therapy [2], [3]. Some patients die and increasing proportions are reported as lost to follow-up (LTFU). However, while many studies have reported on these two outcomes [2]–[7], few have characterised those who transfer-out to other ART services [7]–[9].

We have previously reported from an ART service in South Africa that an increasing proportion of patients enrolling for ART subsequently transfer-out [7]. The characteristics, risk factors and immune and virological status of patients transferring out is unknown. We therefore conducted this detailed analysis of patients transferring out from a large community based ART service in Cape Town, South Africa.

## Materials and Methods

### Setting

The study was conducted at the Hannan Crusaid Treatment Centre in Gugulethu township in Cape Town, Western Cape province, South Africa, which has been described in detail elsewhere [10], [11]. This specialized ART centre is located in a low-income urban area with an antenatal HIV prevalence rate of 24% in 2009 and serviced an estimated population of 366 387 people in 2011 [12]. The gender composition was 48.9% male and 51.1% female. According to census 2011, 19.3% of household heads (n = 95406) were unemployed, 24.4% were not economically active [12]. In general, the Western Cape is the fastest growing province in South Africa, growing by 29% between 2006 and 2011 [12]. This influx of people to the Western Cape highlights the flow of people from rural to urban areas in search of better job opportunities, infrastructure, quality of life and public health services. Among 366 387 people serviced by the clinic, the in-migration streams are predominantly from the Eastern Cape, followed by outside South Africa and then the other provinces [12]. The Eastern Cape, bordering the Western Cape, where much of the migration originates, has a stagnant economy, inferior infrastructure and public health care services. As a result, poor individuals tend to travel back and forth provinces to access better public health care services in the Western Cape.

### Study Cohort

The clinic started providing ART to patients in September 2002 when there were very few other treatment clinics. By the time the national ART roll-out started in April 2004, there were 16 ART sites in the Western Cape province, eight of which were based at primary health care facilities and 2327 patients were then receiving ART [13]. Thereafter, ART scale-up was rapid and by June 2012 there were 121 293 patients receiving ART across 179 sites in the Western Cape province. This nation- and provincial- wide effort to decentralize ART services into more existing primary health care facilities helped to decrease the burden of providing ART services at few initial facilities, made access to ART easier for eligible patients and developed the capacity of clinicians and/or nurses to initiate and monitor treatment [14], [15].

Treatment outcomes at this ART service were recorded in a weekly multidisciplinary meeting and these data were entered in a prospectively maintained ART cohort database on a weekly basis. Patients were classified as alive and on treatment, dead, transferred out or LTFU. ‘Transfer-out’ was the term used to refer to patients who were attending the clinic and whose care was transferred to another clinic by giving the patient a referral letter. A decision to transfer care usually arose as a result of either a new ART service being opened closer to the patient’s home, to relocation of the patient to another area [7] or upon the patient’s request.

Clinic- and field-based counsellors supported the ART service. The counsellors provided educational support, home visits and liaised with the clinical team. At least one home visit was conducted pre-ART and another in the first 4 weeks of ART. Further home visits were conducted for patients classified as defaulting treatment and those with adherence problems. The home visits were, however, optional for patients. Home visits were not conducted for patients who transferred-out as it was assumed that they had resumed treatment at the new facility.

### Study Design

In this retrospective cohort study, all patients (both ART-naïve and non-naïve) aged ≥15 years that were enrolled between September 2002 and December 2009 were included in the analysis. Follow up data were censored in December 2010, providing a minimum of at least one year of potential follow up and a maximum of 8.3 years.

### Data Sources

Data on all patients were obtained from a prospectively maintained ART cohort database. For each patient, clinical variables, outcomes, treatment regimens, and laboratory data were recorded.

### Ethics Statement

Routine data collection in the cohort was undertaken with written informed consent from all patients and ethical approval from the Research Ethics Committee of the University of Cape Town (REC REF 359/2002). Additional ethical approval from the Union’s Ethics Advisory Group (EAG REF: 64/11) was also obtained.

### Study Definitions

‘Patients transferring out’ from this clinic were those who were referred to another facility to continue ART care as documented on a referral form or patient notes. The terms ‘ART-naïve’ and ‘ART non-naïve’ referred to patient status at enrolment to the cohort. The term ‘ART non-naïve’ was used to describe those patients who had previously transferred in to this cohort from another treatment site having already started ART. ‘Virological failure’ was defined as 2 consecutive viral load measurements >1000 copies/mL among patients who had achieved initial virological suppression <400 copies/mL. ‘Second line regimen’ referred to those on TDF/3TC/LPV/r, AZT/3TC/LPV/r and AZT/ddI/LPV/r for adults according to the national ART treatment guidelines 2004 and 2010 [12], [16].

### Statistical Analyses

For the analyses, patients were grouped into those who transferred out and those who did not transfer-out. Categorical variables were described by proportions and differences between patient groups were compared using the χ2 test and Fisher’s exact test, as applicable. Numerical variables were assessed for normality of distribution using Shapiro-Wilk test. Non-normally distributed numerical variables were described by medians and inter-quartile ranges and the differences between groups were compared using Wilcoxon rank-sum test.

Six calendar periods of enrolment were defined as September 2002 to December 2004, followed by 5 calendar periods of 12 months each up to December 2009. The first calendar period combined the first 2 years due to the small numbers of patients enrolled in those years. The probability of transfer-out between patients enrolled in successive calendar periods were estimated using the Kaplan-Meier method, and comparisons were made using the log-rank test. Analyses were stratified a priori according to whether patients were ART-naïve or ART non-naïve since the latter had by definition already transferred in to this cohort from another treatment site and were therefore a highly selected population. Cox proportional hazards analysis was used to determine the risk factors for transferring out. Some risk factors were chosen a priori from our previous research [7], while others included ARV regimen at cohort entry and treatment naive status at baseline. The proportional hazard assumption has been checked graphically using a log–log plot and the Schoenfeld residuals (tests and graphs).

Basic descriptive statistics were used in the analyses of the most recent characteristics prior to transfer. These analyses were restricted to patients who transferred out after at least 1 year of follow-up on ART at this service. A sub-analysis was performed comparing the most recent viral load among ART-naïve patients transferring out with that at a comparable time point among ART-naïve patients not transferring out. A folder review was also conducted to determine the reason why some ART-naïve patients transferred out with a viral load ≥1000 copies/mL.

Wald confidence limits were used for all multivariate models. All statistical tests were 2-sided at alpha of 0.05. Stata statistical software, version 10.0 was used for analyses (Stata Corporation, College Station, TX).

## Results

### Patients and Follow-up

**Figure 1:**
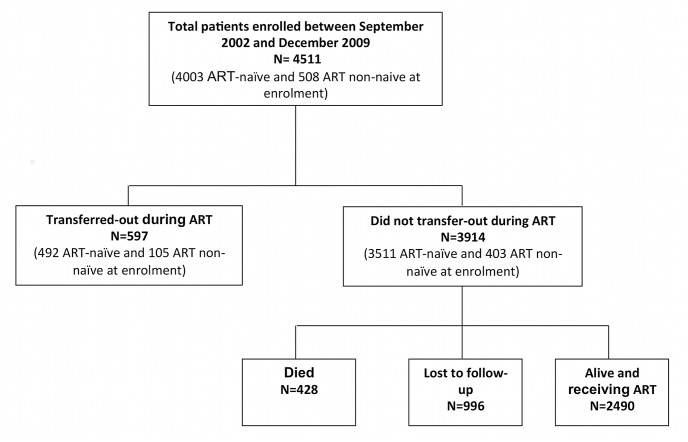
Description of patients starting ART at a community-based ART service in Gugulethu, Cape Town between September 2002 and December 2009.

Between September 2002 and December 2009, a total of 4511 patients received ART (**Figure 1**). At baseline a majority (88.7%) were ART-naïve, the majority were female (67.2%) and the median age was 29.0 years (interquartile range [IQR], 15.0–33.6). Immunodeficiency was advanced with a median blood CD4 lymphocyte count of 109 cells/µL (IQR, 53–168). Disease was categorized as WHO clinical stage III or IV in 73.1%.

The median duration of follow-up on ART was 2.5 years (IQR 1.2–4.3), with a minimum of 1.0 year and a maximum of 8.3 years. A total of 12699 person-years of follow-up accrued during the analysis period during which 597 (13.2%) patients transferred out. In addition, 428 (9.5%) died, 996 (22.1%) were LTFU and 2490 (55.2%) were alive and receiving ART at the time data were censored (**Figure 1**).

### Rates and Proportions Transferring Out

**Figure 2:**
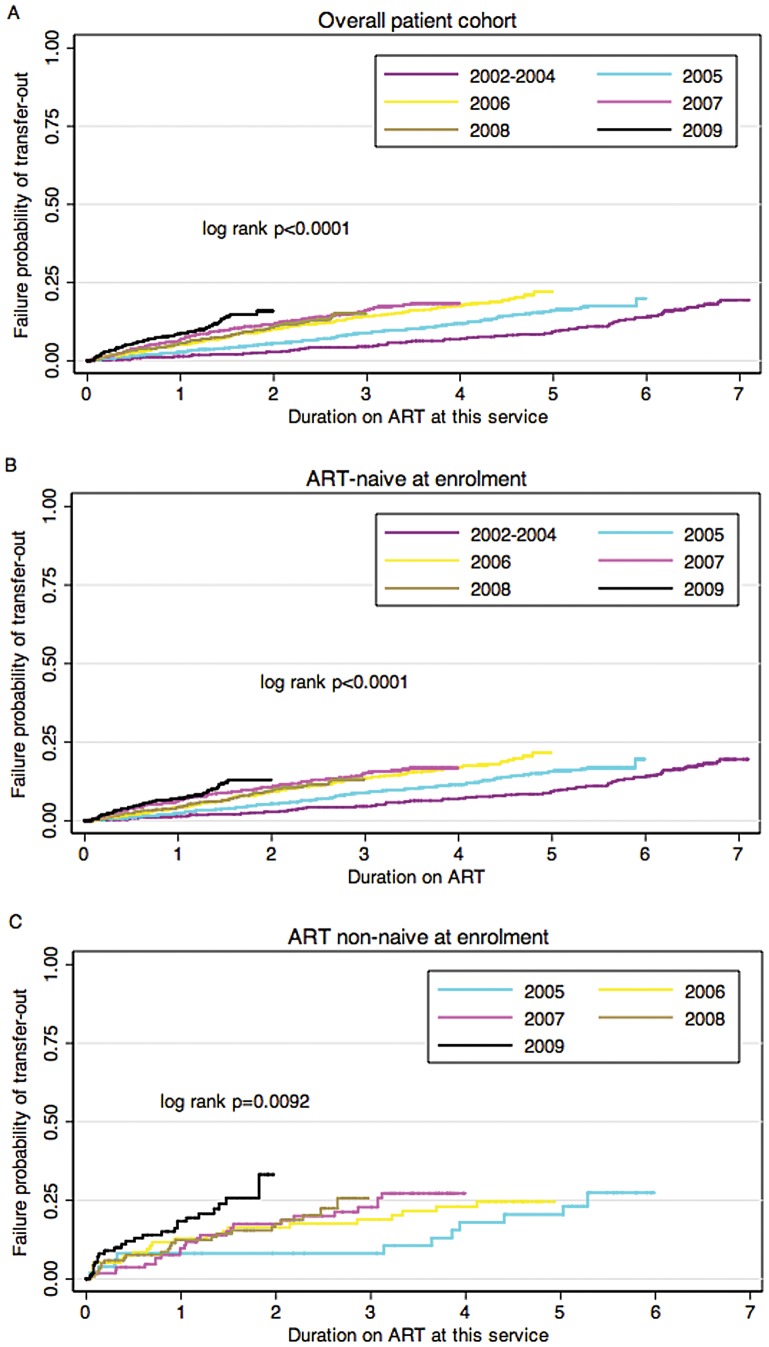
Failure probability of transferring out by calendar period of enrolment a) overall patient cohort, b) among ART-naïve and c) ART non-naive patients attending a community-based clinic in Cape Town, South Africa.

Overall, the rate of transfer-out was 4.7 (95% CI 4.3–5.1) patients per 100 person-years. The transfer-out rate was higher among ART non-naïve patients (9.11 per 100 person-years) compared to those who were ART-naïve (4.24 per 100 person-years; P<0.0001). We used Kaplan-Meier analyses to examine how the proportion of patients transferred out varied by calendar period of enrolment. The proportion transferred-out increased with more recent calendar periods of enrolment (**Figure 2A**). Overall, the probability of transfer-out at 1 year increased from 1.4% (95% CI 0.6%–3.0%) in the 2002–2004 cohort to 8.9% (95% CI 4.3%–7.8%) in the 2009 cohort. Similar trends were observed in analyses stratifying by baseline ART status (**Figure 2B** **and** **Figure 2C**), although overall proportions were higher among the ART-non-naïve patients. Overall, the probability of transfer-out at 1 year was 18.3% (95% CI, 12.1–27.3) among non-naïve patients compared to 7.2% (95% CI, 5.4–9.5) among ART-naïve patients (P<0.01).

### Characteristics of Patients Transferring Out

We next examined the characteristics of patients who did and those who did not transfer-out, stratifying by baseline ART status. Among those who were ART-naïve at baseline, patients who transferred out were more likely to be female and younger (**Table 1**). Among those who were ART non-naïve at baseline, the characteristics of those who did and did not transfer-out were broadly similar. An important observation was that of the non-naïve patients (ie those who transferred into the service already receiving ART), only 69.4% had a viral load suppressed <50 copies/mL and only 78.8% had a viral load suppressed <400 copies/mL (**Table 1**).

**Table 1:** Baseline characteristics of patients who transferred out and those who did not transfer-out from a community-based ART service in Gugulethu, Cape Town between September 2002 and December 2009.

	ART-naïve patients at enrolment N = 4003				ART non-naïve at enrolment N = 508			
Variable	Patients transferring out		Patients not transferring out		Patients transferring out		Patients not transferring out	
	N = 492		N = 3511		N = 105		N = 403	
	N	% (95% CI)	N	% (95% CI)	N	% (95% CI)	N	% (95% CI)
**Female**	346	70.3 (66.3–74.3)	2312	65.9 (64.3–67.4)	81	77.1 (69.1–85.2	291	72.2 (67.8–76.6)
**Age at start of ART (yrs),** **median (IQR)**	33.0 (28.5–39.1)	–	33.8 (29.1–40.2)	–	32.0 (27.7–39.1)	–	32.8 (28.4–38.0)	–
**CD4 at cohort entry (cells/µL)** * **,** **median (IQR)**	105 (48–158)	–	109 (55–168)	–	328 (235–442)	–	312 (190–457.5)	–
**CD4 cell count category at** **cohort entry** * **(cells/µL)**								
**≤100**	240	49.1 (44.6–53.5)	1570	46.1 (44.4–47.7)	3	3.5 (−0.4–7.5)	34	10.1 (6.9–13.3)
**101–200**	192	39.3 (34.9– 43.6)	1374	40.3 (38.7–42.0)	14	16.5 (8.6–24.4)	61	18.2 (14.0–22.3)
**>200**	57	11.7 (8.8–14.5)	464	13.6 (12.5–14.8)	68	80.0 (71.5–88.5)	241	71.7 (66.9–76.5)
**WHO stage at ART initiation**								
**stage 1 and II**	124	25.3 (21.4–29.1)	963	27.5 (26.0–28.9)		–	–	–
**stage III and IV**	367	74.8 (70.9–78.6)	2545	72.6 (71.1–74.0)		–	–	–
**Viral load at cohort entry** † **(copies/mL)**								
**Log_10_ median (IQR)**	4.9 (4.5–5.4)	–	4.9 (4.4–5.3)	–	1.7 (1.7–2.1)	–	1.7 (1.7–2.3)	–
**Viral load at cohort entry** †								
**<50**	–	–		–	59	69.4 (59.6–79.2)	223	66.8 (61.7–71.8)
**<400**	–	–		–	67	78.8 (70.1–87.5)	260	77.8 (73.4–82.3)
**<1000**	–	–		–	73	85.9 (78.5–93.3)	274	82.0 (77.9–86.2)
**Regimen**								
**EFV-based**	306	62.0 (57.7–66.3)	2162	61.7 (60.1–63.3)	69	68.3 (59.2–77.4)	257	65.6 (60.9–70.3)
**NVP- based**	186	37.8 (33.5–42.1)	1338	38.2 (36.6–39.8)	29	29.7 (.2079137.3861457)	123	31.4 (.2678401.3597109)
**PI-based**	1	0.2 (−0.2–0.6)	6	0.2 (0.03–0.3)	2	2.0 (−0.7– 4.7)	12	3.1 (−1.4– 4.7)

CI, confidence interval.

* Data were available for 97.4% (3897/4003) ART naïve patients and 82.8% (421/508) non-naïve patients.

† Data were available for 96.9% (3878/4003) ART naïve patients and 82.5% (419/508) non-naïve patients.

### Risk Factors for Transfer Out

We used Cox proportional hazards analysis to examine the risk factors associated with transferring out (**Table 2**). In crude analyses, risk (or hazard) of transfer-out was associated with calendar year of enrolment, young age and being ART non-naïve at baseline. There was also an association with baseline CD4 cell count and viral load although these were also strongly associated with baseline ART status. In multivariate analysis, increased risk of transfer-out was strongly associated with more recent calendar period of enrolment and being ART non-naïve at enrolment (P<0.001) (**Table 2**).

**Table 2:** Baseline patient characteristics associated with transferring out in a Multivariate Cox proportional Hazard Model.

Variables	Unadjusted HazardRatio (95% CI)	p-value	Adjusted HazardRatio (95% CI)	p-value
**Calendar period of enrolment**				
**Sept 2002–Dec 2004 (Ref)**	1.00	–	1.00	–
**Jan–Dec 2005**	1.54 (1.10–2.16)	0.012	1.46 (1.04–2.07)	0.030
**Jan–Dec 2006**	2.41 (1.72–3.39)	<0.001	2.32 (1.64–3.28)	<0.001
**Jan–Dec 2007**	2.86 (2.01–4.08)	<0.001	2.58 (1.78–3.74)	<0.001
**Jan–Dec 2008**	2.81 (1.93–4.08)	<0.001	2.60 (1.76–3.84)	<0.001
**Jan–Dec 2009**	4.46 (3.05–6.51)	<0.001	4.00 (2.69–5.98)	<0.001
**Gender**				
**Female (ref)**	1.00	–	1.00	–
**Male**	0.90 (0.75–1.07)	0.231	0.89 (0.73–1.08)	0.223
**Age group**				
**15**–**25**	1.41 (1.06–1.88)	0.019	1.41 (1.04–1.91)	0.026
**26**–**30**	1.07 (0.86–1.37)	0.528	1.11 (0.86–1.42)	0.437
**31**–**40**	0.95 (0.77–1.17)	0.603	0.94 (0.76–1.17)	0.569
**>41**	1.00	–	1.00	–
**Regimen at cohort entry**				
**EFV-based (ref)**	1.00	–	1.00	–
**NVP- based**	1.13 (0.95–1.34)	0.156	0.93 (0.77–1.13)	0.463
**PI-based**	1.38 (0.44–4.29)	0.583	0.84 (0.27– 2.64)	0.762
**Treatment experience**				
**ART-naïve (ref)**	1.00	–	1.00	–
**ART non-naïve**	2.12 (1.71–2.62)	<0.001	1.96 (1.38–2.78)	<0.001
**CD4 count at cohort entry**				
**≤100**	1.00	–	1.00	–
**101**–**200**	0.91 (0.76–1.10)	0.347	0.88 (0.73–1.07)	0.204
**≥201**	1.32 (1.06–1.64)	0.012	0.95 (0.73–1.24)	0.719
**Viral load at cohort entry**				
**<4**	1.00	–	100	–
**4**–**4.9**	0.72 (0.57–0.90)	0.003	1.11 (0.82–1.49)	0.498
**≥5**	0.83 (0.66–1.03)	0.090	1.28 (0.94–1.74)	0.116

CI, confidence interval.

### Patient Characteristics at Time of Transfer Out

We next examined the characteristics of patients just prior to transfer-out. We restricted this analysis to those transferring out after at least one year of follow-up on ART to allow time for virological suppression. The median duration on ART at the time of transfer-out was 29.0 months (IQR 19.1–45.6) among patients who were ART-naïve at baseline compared to 24.7 months (IQR 17.3–37.4) among those who were non-naïve at baseline (**Table 3**). The proportions of these two groups of patients with CD4 cell counts <200 cells/µL were 20.1% and 13.4%, respectively. The proportion of ART-naïve and non-naïve patients transferred out with a viral load ≥1000 copies/mL was 20.7% and 15.6%, respectively.

**Table 3:** Characteristics at the time of transfer for both ART-naïve and ART non-naive patients from a community-based ART service in Gugulethu, Cape Town between September 2002 and December 2009.

	Overall	ART-naïve at enrolment	ART non-naïve at enrolment
	N = 375	N = 330	N = 45
**Variable**		Patients transferring out	Patients transferring out
**Time on ART at this service (months), median (IQR)**	28.5 (18.6–44.4)	29.0 (19.1–45.6)	24.7 (17.3–37.4)
**CD4 count at time of transfer (cells/µL)** *			
**≤100**	24 (6.4)	21 (6.4)	3 (6.7)
**101**–**200**	48 (12.8)	45 (13.7)	3 (6.7)
**>200**	302 (80.8)	263 (79.9)	39 (86.7)
**Viral load at the time of transfer (copies/mL), n (%)** †			
**≥50**	107 (28.6)	96 (29.0)	11 (24.4)
**≥400**	84 (22.5)	76 (23.2)	7 (15.6)
**≥1000**	76 (20.3)	68 (20.7)	7 (15.6)
**Virological failure at the time of transfer, n (%)**			
**Never**	311 (82.9)	269 (81.5)	42 (93.3)
**At any time point**	35 (9.3)	61 (18.5)	3 (6.7)
**≤1 year**	29 (7.7)	35 (10.6)	0 (0.0)
**>1 year**		26 (7.9)	3 (6.7)
**Regimen at the time of transfer, n (%)**			
**First line**	327 (88.1)	289 (88.7)	38 (84.4)
**Second line**	44 (11.9)	37 (11.4)	7 (15.6)
**Place of transfer**			
**Cape Town**	222 (59.2)	195 (59.1)	27 (60.0)
**Other**	153 (40.8)	135 (40.9)	18 (40.0)

All analyses restricted to patients that transferred out with at least 1 year of follow-up on ART at this service. This was done to allow sufficient time for virological suppression as well as for blood samples to be drawn at most 3 times for CD and VL measurements.

* CD4 cell count data at the time of transfer were available for 329 ART naïve and 45 ART non naïve patients.

† Viral load data at the time of transfer were available for 329 ART naïve and 45 ART non-naïve patients.

We next performed a sub-analysis restricted to those who were ART-naïve at baseline and compared the most recent viral loads of patients who did and did not transfer-out after a similar ART duration. Those transferring out were more likely to have a viral load ≥1000 copies/mL in both unadjusted and adjusted analyses (unadjusted risk ratio 1.72, 95% CI 1.29–2.30; adjusted risk ratio 1.67, 95% CI 1.24–2.24). Further investigation found that 44.1% (95% CI, 32.0%–56.2%) of ART-naïve patients transferring out with a viral load ≥1000 copies/mL had not collected ART ≤12 weeks before the transfer-out date.

We then analyzed the viral loads of patients who were ART non-naïve at baseline and compared virological suppression rates at baseline and at the time of transfer-out. Of those who had a viral load ≥1000 copies/mL when they were transferred in [11.4% (95% CI, 5.2%−17.6%)] (**Table 1**), 66.7% (95% CI, 35.4%−98.0%) of them subsequently transferred out with a viral load ≥1000 copies/mL. Among this group, 50% (95% CI, 5.3%−94.7%) were on first line treatment and the remaining 50% (95% CI, 5.3%–94.7%) were on second-line treatment at the time of transfer-out.

## Discussion

In this large cohort study, we found that overall 13.2% of patients transferred out during a median follow-up of 2.5 years but that the proportion increased substantially with more recent calendar periods of enrolment. Additional risk factors for transfer-out were younger age (≤25 years) and being non-naïve to antiretroviral therapy at baseline. A high proportion of patients (20%) did not have a suppressed viral load just prior to transfer and there was evidence that discontinuity of ART was common around the time of transfer. Moreover, of patients on ART transferring in to this service from elsewhere, many had detectable viral loads, again suggesting discontinuity of medication supplies or poor adherence.

Little is known about transfer-out rates in ART programmes in sub-Saharan Africa. A study from Botswana reported a transfer out rate of 5.2% after 1 year [9]. Our study documented a steady increase in the proportion of patients transferring out, with this proportion increasing from 1.4% to 8.9% at one year when comparing the 2002–2004 cohort with the 2009 cohort. The reasons for this phenomenon have not previously been documented. This is likely to reflect the availability of ART at increasing numbers of primary health care centres nearer to patients’ homes and increasing confidence and diminishing stigma among individuals living with HIV, resulting in increased social acceptance and mobility. Other explanations may be that patients wished to save on transport and time by attending facilities closer to their homes [16] and that increasing congestion [7] and depersonalization at this large clinic may have lowered the threshold for patients requesting transfer-out. The rate of transfer-out may have been further catalyzed by the policy of decentralization of ART to a wider network of primary health care centres. The scale up of provider initiated counseling and testing by the national TB program in 2005 [17], the implementation of the national HIV counseling and testing policy guidelines in 2010 [18] and the change in national ART guidelines from an ART eligibility of CD4 count 200 cells/µL to 350 cells/µL in early 2010 [19] all resulted in increased numbers of individuals accessing ART, requiring further decentralisation. Lastly, this finding may be due the out- migration of patients in the clinic mainly originating from the Eastern Cape who had come to Cape Town in search of better ART services, job opportunities, infrastructure and quality of life.

The escalating rates of transfer-out over time presents a challenge to the health care system and to accurate documentation of outcomes and programme performance. There is a need for reliable tracking systems that can ascertain whether transfers of care have been successful. Although such patient tracking systems exist in some sites offering ART, there are yet to be implemented across sites on a country-wide basis (including at our ART service). Moreover, the implementation of such patient tracking systems in many other sub-Saharan countries has been challenging [2].

Younger age and being non-naïve to ART at baseline were associated with higher risk of transferring out. It is possible that younger people attending the clinic are more likely to move to other areas in search of employment, better opportunities and better social support networks. Opportunity, poverty or poor social support networks may therefore dictate transfer-out. Our data suggest that a proportion of those patients who were non-naïve to ART at baseline (ie those who had already transferred out from another clinic and enrolled in this clinic) serially transfer between clinics and that a significant proportion had unsuppressed viral loads. These may represent an important sub-group of patients with poor adherence, which may have important underlying factors such as social stigma and non-disclosure.

Achieving and sustaining viral suppression is key in the delivery of long-term ART. Our findings are of real concern that a substantial proportions of patients had viral loads >1000 copies/mL around the time of both transfer-out and transfer-in to this ART service. This could be due to patients who move or gain employment elsewhere and who subsequently struggle to attended scheduled appointments, before eventually requesting transfer to another facility. This may also indicate poor adherence, with treatment interruption often occurring prior to the date of transfer. Alternatively, this could indicate high rates of viral resistance (transmitted or acquired). Studies have shown that factors such as ART adverse effects, substance abuse, clinical depression and clinicians lack of experience to treat HIV disease are all associated with increased risk of an unsuppressed viral load [12], [20]–[23]. The specific factors relevant in this cohort need to be understood through further research and appropriate interventions need to be identified and implemented. From our own experience, we speculate that intensive counselor-driven interventions addressing poor adherence, including addressing social-related factors (i.e. substance abuse, clinical depression, encouraging patients to join social support groups) may be necessary.

The strengths of the study include the study of a very well characterized cohort with careful patient follow-up, including 4-monthly CD4 cell count and viral load measurements. The patient within this township cohort are typical of large public sector ART cohorts throughout Cape Town, South Africa. Study limitations include selection bias due to missing information in multivariate analysis may exist although this is likely to be minimal in view of the completeness of the data collected and that the few missing observations were random. This was an observational study that used data collected from routine program evaluation which are limited by operational constraints. The contribution of socioeconomic status of ART patients on transfer out rates could not be ascertained from our data. However, the community in which the clinic operated has a high unemployment rate in general and therefore serves mainly low-income ART patients. This manuscript does not deal with silent or unnoticed transfers-out and this may underestimate our finding of increased transfer-out with yearly cohorts. We were not able to ascertain the final outcomes of patients who transferred out from this service.

### Conclusions

In summary, during ongoing scale-up of ART, increasing proportions of patients are transferring between ART services and the reasons for this are not yet well understood. It is of real concern that approximately one fifth of patients had viral loads of >1000 copies/mL around the time of transfer, suggesting the need for careful adherence counseling and assessment of medication supplies among those planning to transfer. Furthermore, information systems need to be developed that can track patients, to assess successful transfer of care between health care facilities and to provide accurate programme outcome data.
